# Transcatheter aortic valve replacement for aortic regurgitation following valve sparing root replacement: a case series

**DOI:** 10.1093/ehjcr/ytae674

**Published:** 2024-12-23

**Authors:** Harish Sharma, Anthony Mechery, Ewa Lawton, M Adnan Nadir, Sagar N Doshi

**Affiliations:** Institute of Cardiovascular Sciences, University of Birmingham, Birmingham B15 2TT, UK; Department of Cardiology, Queen Elizabeth Hospital Birmingham, Birmingham B15 2TH, UK; Department of Cardiology, Queen Elizabeth Hospital Birmingham, Birmingham B15 2TH, UK; Department of Cardiology, Queen Elizabeth Hospital Birmingham, Birmingham B15 2TH, UK; Institute of Cardiovascular Sciences, University of Birmingham, Birmingham B15 2TT, UK; Department of Cardiology, Queen Elizabeth Hospital Birmingham, Birmingham B15 2TH, UK; Institute of Cardiovascular Sciences, University of Birmingham, Birmingham B15 2TT, UK; Department of Cardiology, Queen Elizabeth Hospital Birmingham, Birmingham B15 2TH, UK

**Keywords:** Transcatheter aortic valve replacement, Valve sparing aortic root replacement, Case report

## Abstract

**Background:**

Valve sparing aortic root replacement (VSARR) is a treatment for aortic root dilatation and aortic regurgitation (AR), which preserves the aortic valve. However, AR may recur, and redo surgery often carries high risk. Transcatheter aortic valve replacement (TAVR) can be performed but there is a paucity of literature to guide procedural planning.

**Case summary:**

Two cases are presented herein with recurrence of severe AR following VSARR (David procedure). In both cases, computed tomography (CT) scans demonstrated absence of calcium and a narrow sinus of valsalva. Both cases were considered at prohibitive risk for redo surgery and were successfully treated with TAVR using balloon-expandable valves. The valves were sized based on CT (aiming for moderate oversizing of 10%–11%) and by assessing the anchoring and waist of sizing balloons. Post-procedure aortography and echocardiography revealed no transvalvular or paravalvular regurgitation. Both patients were successfully discharged with follow-up CT scans also showing no migration and preservation of coronary access, together with suitability of future redo TAVR if required.

**Discussion:**

TAVR can be successfully performed after VSARR surgery with a balloon-expandable valve in the absence of aortic valve calcification. Moderate THV oversizing (10%–11%) appears safe and effective. Careful assessment with a sizing balloon is recommended to ensure adequate anchoring, without the need for aggressive oversizing which risks rupture at the graft suture line.

**Learning points**

- Persistence or recurrence of aortic regurgitation following valve sparing aortic root replacement (VSARR) poses a challenge for treatment by transcatheter aortic valve replacement (TAVR) due to the absence of native leaflet calcification and distensibility of the graft.
- Balloon-expandable transcatheter heart valves with modest oversizing (10%–11%) can be used to successfully manage pure AR occurring in VSARR failure. Sizing can be further guided by the stability and waisting of a valvuloplasty balloon.
- Short-framed balloon-expandable valves may be preferable in view of better coronary access, particularly in younger patients who may require redo TAVR and cases of low left main stem height.

## Introduction

Aortic root dilatation, without significant aortic valve disease, can be treated by valve sparing aortic root replacement (VSARR), whereby the aortic root is replaced with a Dacron graft, reimplanting the native coronary arteries, and sparing the native aortic valve, which is resuspended within the graft. In the late 1970s, Sir Magdi Yacoub described a technique, which involves ‘remodelling’ the aortic root annulus by replacing the sinus of valsalva with a 3 scalloped polyester graft that preserved the aortic valve but did not protect the annulus from dilatation.^1^ 10 years later Dr. Tirone David and colleagues introduced the reimplantation operation whereby a Dacron graft is seated below the aortic valve resulting in protection of the valve leaflets and annulus.^2^

VSARR has gained popularity, as it avoids anticoagulation and obviates the complications of prosthetic valves such as patient-prosthesis mismatch, thromboembolism and structural valve degeneration. Although VSARR is associated with low rates of mortality,^3,4^ aortic regurgitation (AR) may persist or recur^5–8^ with re-intervention required in up to 10% within 8 years.^4,9^ Furthermore, AR frequently develops in the absence of significant leaflet calcification. Redo surgery in these patients carries high procedural risk and transcatheter aortic valve replacement (TAVR) is conceptually appealing due to its limited invasiveness and lower procedural risk. However, TAVR is made challenging due to the frequent lack of leaflet calcification, associated risk of paravalvular regurgitation and risk of annular rupture at the graft/left ventricular outflow tract (LVOT) suture line. Absence of leaflet calcification additionally carries the serious risk of valve migration/embolization. Currently, the only transcatheter heart valve (THV) licensed in Europe for use in pure AR with absent leaflet calcification is the Trilogy (JenaValve Technology, California, USA). However, only three sizes are currently available with largest (27 mm) accommodating an annulus of up to 90 mm perimeter. Although off-label use of THVs have been successfully used to treat aortic stenosis and regurgitation following VSARR, reports are few in number and there is little guidance on the choice of THV and optimal sizing strategy.^10–13^ We present a case series providing insights into how TAVR may be performed successfully in such cases with a balloon-expandable valve and provide recommendation on a sizing strategy.

## Summary figure

**Figure:**
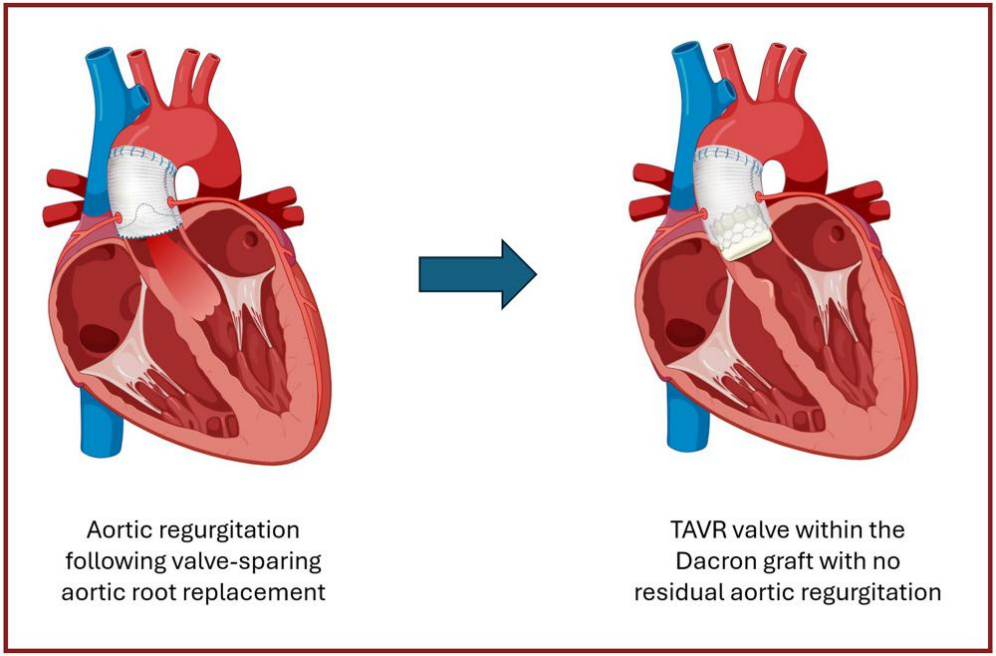


## Patient 1

A 68-year-old female underwent VSARR (David) with a 30 mm Vascutek Valsalva graft (Terumo, Tokyo, Japan) and surgical AF ablation in 2020 for aortic root dilatation (59 mm diameter sinus of Valsalva) and moderate AR (vena contracta 0.6 cm, pressure half-time 484 ms). Pre-operative echocardiography demonstrated the native aortic valve was trileaflet with mild leaflet thickening but no calcification and left ventricular (LV) function was impaired (45%). Coronary angiography showed no significant coronary artery disease. Post-operatively, she suffered a reduction in cardiac output with coronary angiography now revealing iatrogenic ostial stenosis of the right coronary artery (RCA) caused during reimplantation of the right coronary button. Repeat sternotomy was performed with a saphenous vein graft to the RCA. Post-operative transthoracic echocardiography (TTE) revealed severely impaired LV function (LVEF 30%) and moderate AR.

**Figure 1:**
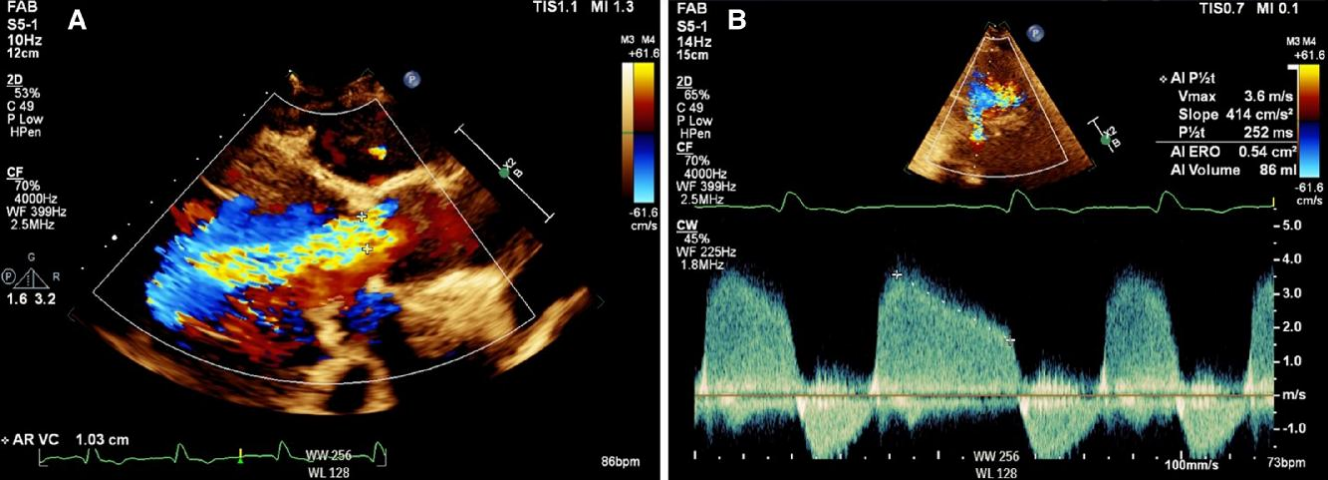
Transthoracic echocardiography demonstrating severe aortic regurgitation. (*A*) Parasternal long axis (vena contracta 1 cm); (*B*) continuous wave (CW) Doppler through the aortic valve in the apical 5-chamber view.

**Figure 2:**
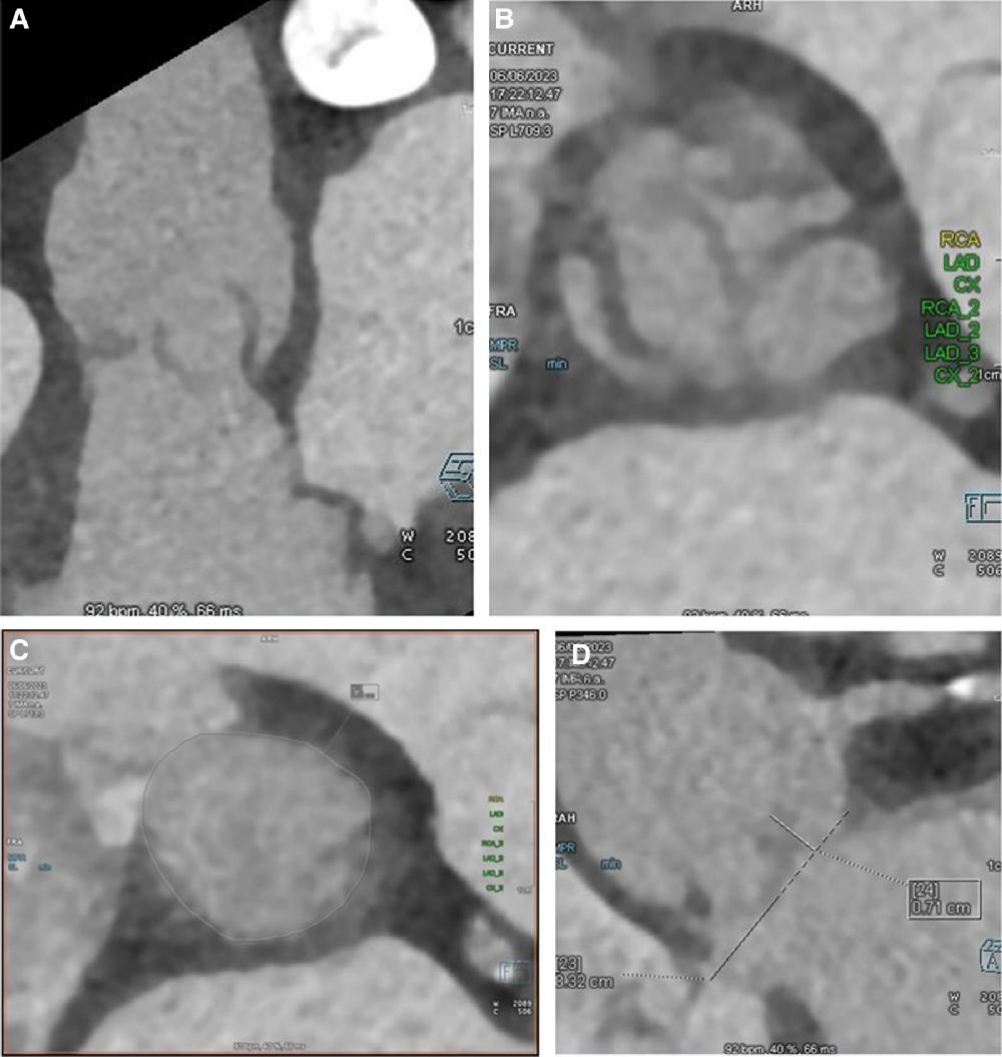
Computed tomography demonstrating absence of aortic valve calcification (*A* and *B*). (*C*) Neo-annulus measured 660 mm^2^; (*D*) left main coronary height 7.1 mm.

In 2023, she re-presented with dyspnoea and TTE demonstrated severe AR with poor LV function (LVEF 15%) (*Figure 1*). Computed tomography (CT) demonstrated absence of aortic valve calcification. The neo-annulus measured 660 mm^2^ and left coronary height was low at 7.1 mm (*Figure 2*). The sinus of valsalva was relatively narrow at 31 mm (mean). The sinuotubular junction (STJ) was also narrow (28 mm) with a height of 24 mm. Redo surgery was felt to carry high risk and the Heart Team consensus was for TAVR. The annulus was too large for the 27 mm Trilogy valve. A balloon-expandable THV was chosen in view of its good coronary access and suitability for redo TAVR.

**Figure 3:**
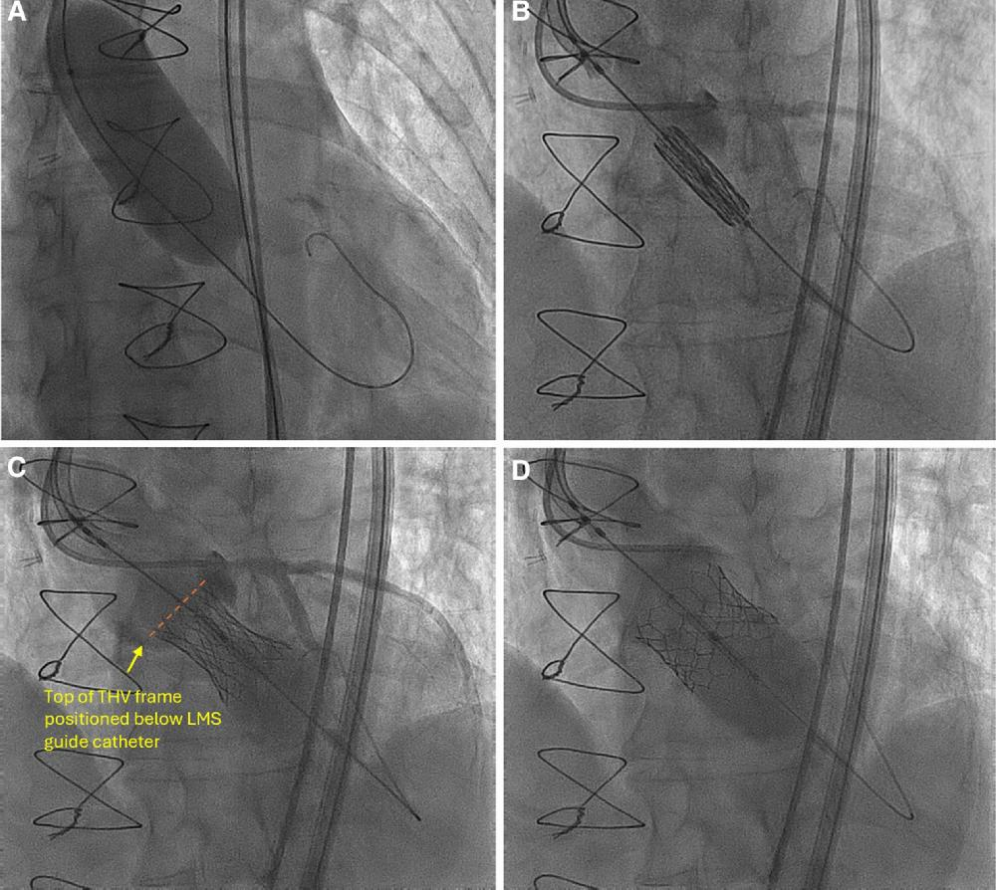
Fluoroscopy demonstrating a lack of balloon waisting (*A*), positioning (*B*), and deployment (*C* and *D*) of the transcatheter heart valve (THV) below the left main coronary artery.

**Figure 4:**
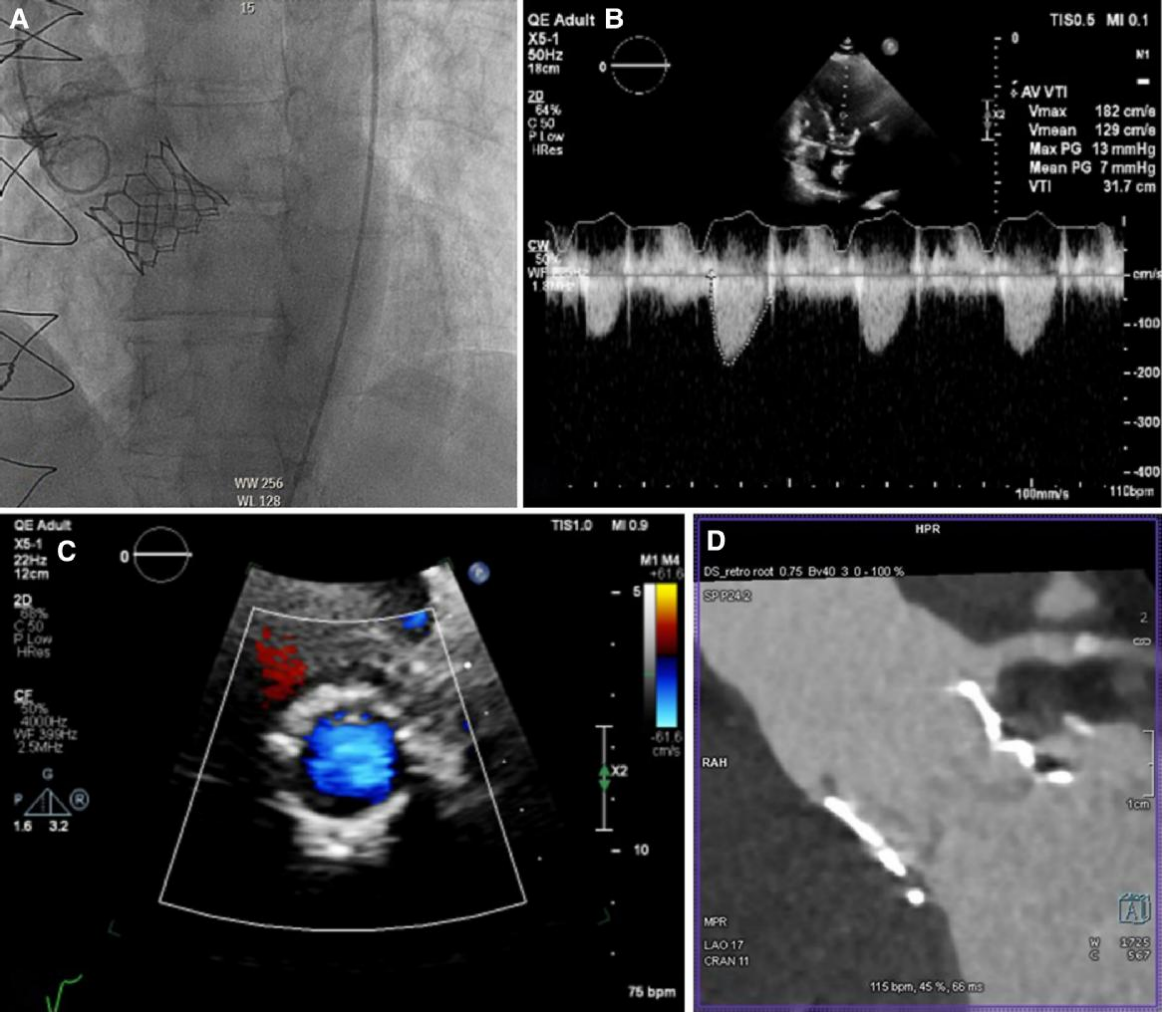
(*A*) Post-transcatheter aortic valve replacement aortography showed no aortic regurgitation: (*B*) continuous wave Doppler through the aortic valve; (*C*) colour Doppler in the short axis view; (*D*) computed tomography at follow-up showed preservation of left main coronary access.

In July 2023, transfemoral TAVR was performed. To help guide THV sizing, a 28 mm Cristal Balloon (Balt, Montmorency, France) was inflated in the neo-annulus during burst pacing. At full inflation, the balloon anchored well, but notably had no waist. In light of stability of the 28 mm Cristal balloon, but absence of a waist, a 29 mm Sapien 3 (Edwards Lifesciences, California, USA) was overfilled (3 cc) to achieve an approximate area of 730 mm^2^ (estimated oversizing 11%). A left coronary guide catheter was engaged to demarcate the left main coronary artery (LMCA) to aid positioning of the THV. A wire was passed into the left anterior descending artery (LAD) with a coronary balloon placed over the wire within the LAD to ensure stability of the guide catheter. The THV was then positioned below the LMCA in order to preserve unrestricted coronary access and facilitate future redo TAVR (*Figure 3*). Post-TAVR aortography showed no AR (*Figure 4*) and only a mild waist of the THV. A pacemaker was implanted for complete heart block post-procedure. At 3 months a TTE showed a well-functioning THV with no transvalvular or paravalvular AR, aortic valve area 2.2 cm^2^, and mean gradient 7 mmHg. CT imaging at 3 months showed no THV migration and a position below the level of the left coronary ostium (*Figure 4*). LV function remained poor and the pacemaker has been upgraded to a CRT device.

## Patient 2

**Figure 5:**
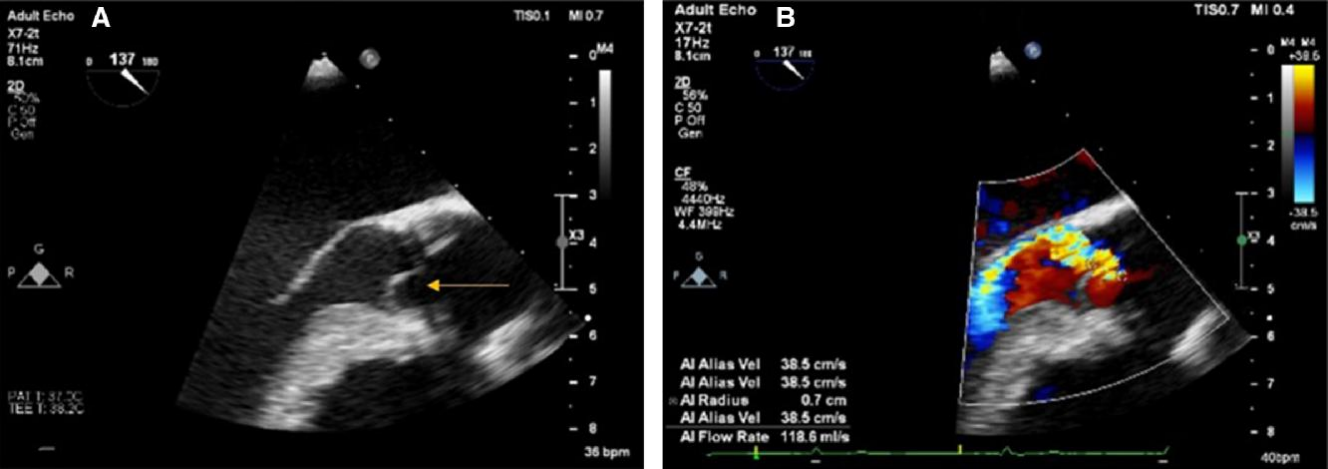
Transoesophageal echocardiography (long axis view) demonstrating recurrence of aortic regurgitation (AR) after valve sparing aortic root replacement due to prolapse of the right coronary cusp (arrow—*A*) and with colour Doppler (*B*) showing an eccentric jet of severe AR (vena contracta 0.7 cm).

**Figure 6:**
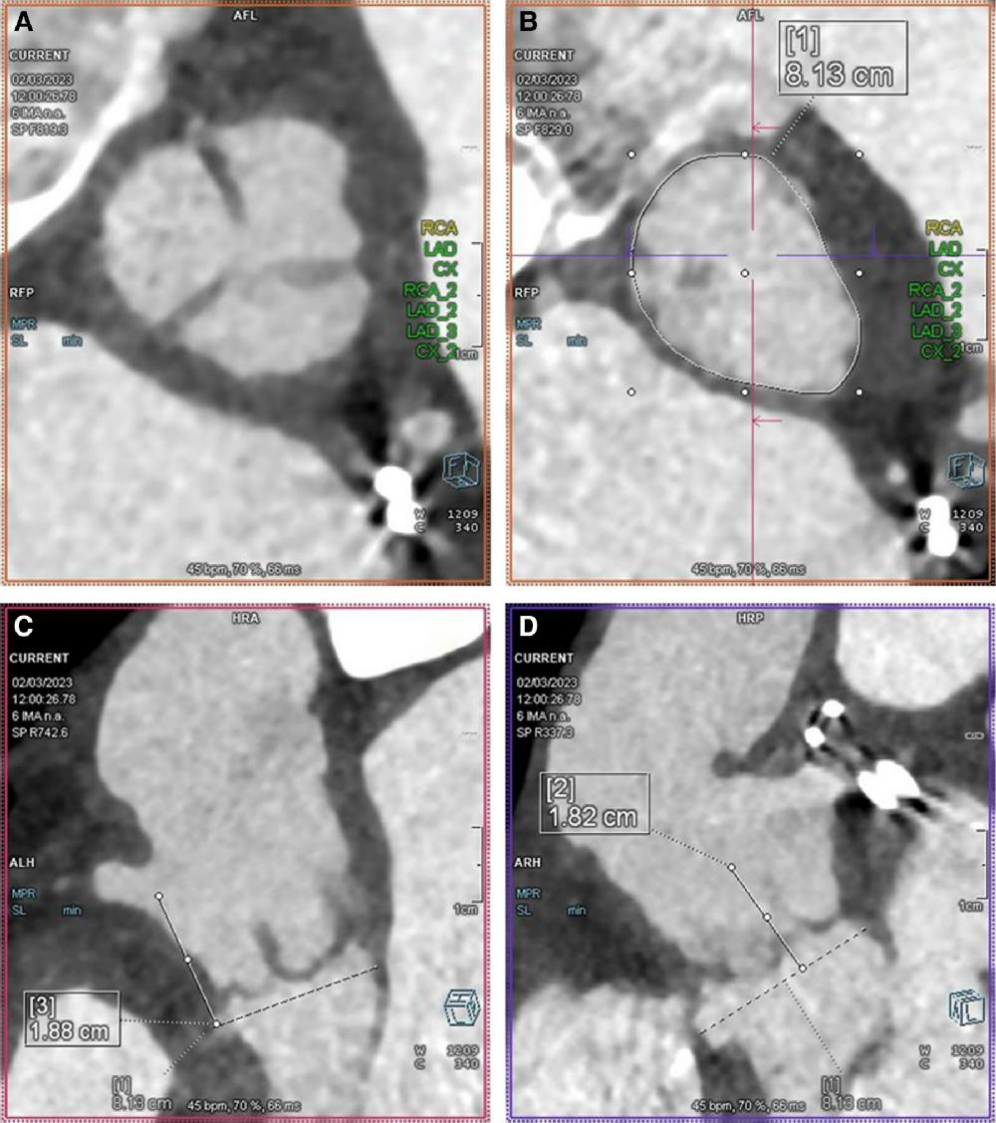
Computed tomography scan showing absence of leaflet calcification (*A*), a perimeter of 8.13 cm (*B*), right coronary artery height of 18.8 mm (*C*), and left main coronary height of 18.2 mm (*D*).

A 71-year-old man with aortic root dilatation and severe AR underwent VSARR (David) in 2021 using a 26 mm Vascutek Gelwave Valsalva graft (Terumo) with coronary reimplantation. The aortic valve was tricuspid and not calcified. Post-operative TTE showed normal LV size and function with mild AR. In 2022, he presented with progressive dyspnoea and peripheral oedema. Transoesophageal echocardiography revealed severe AR due to prolapse of the right coronary cusp (*Figure 5*). The LV was moderately dilated with good systolic function (LVEF 67%). Redo surgery was felt to carry high risk and the Heart Team consensus was for TAVR. The neo-annulus measured 525 mm^2^, LMCA height 18.2 mm, and RCA height 18.8 mm (*Figure 6*). The sinus of valsalva and STJ were narrow (28 and 25 mm) and there was a ‘kink’ at the STJ (*Figure 6D*). STJ height was 21 mm. Due to lack of availability of the Trilogy JenaValve, transfemoral TAVR was planned with a 26 mm Edwards Sapien Ultra valve.

**Figure 7:**
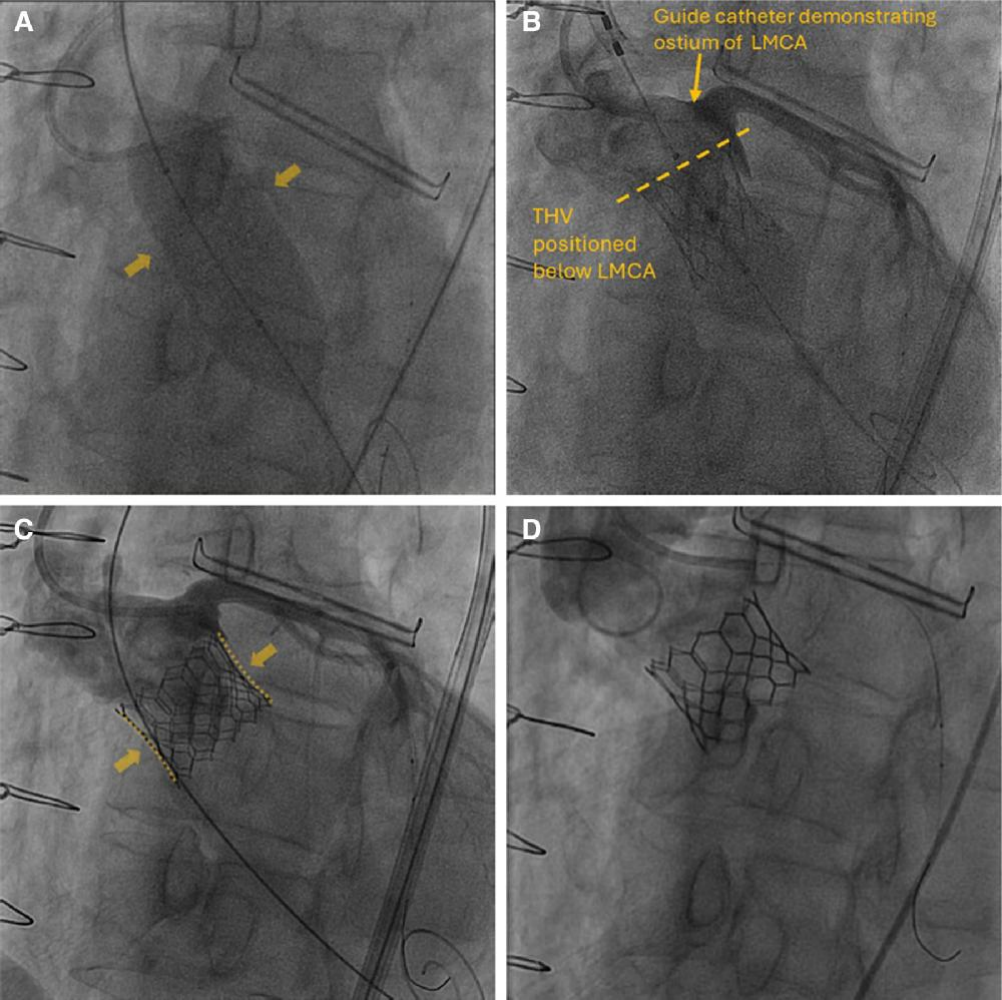
A 25 mm sizing balloon anchored (*A*). The 26 mm Sapien transcatheter heart valve (THV) was positioned below the left main coronary artery and deployed with 4 cc overfill (*B*). The mild waist of the THV frame indicates good anchoring (*C*) with no residual aortic regurgitation on aortography (*D*).

**Figure 8:**
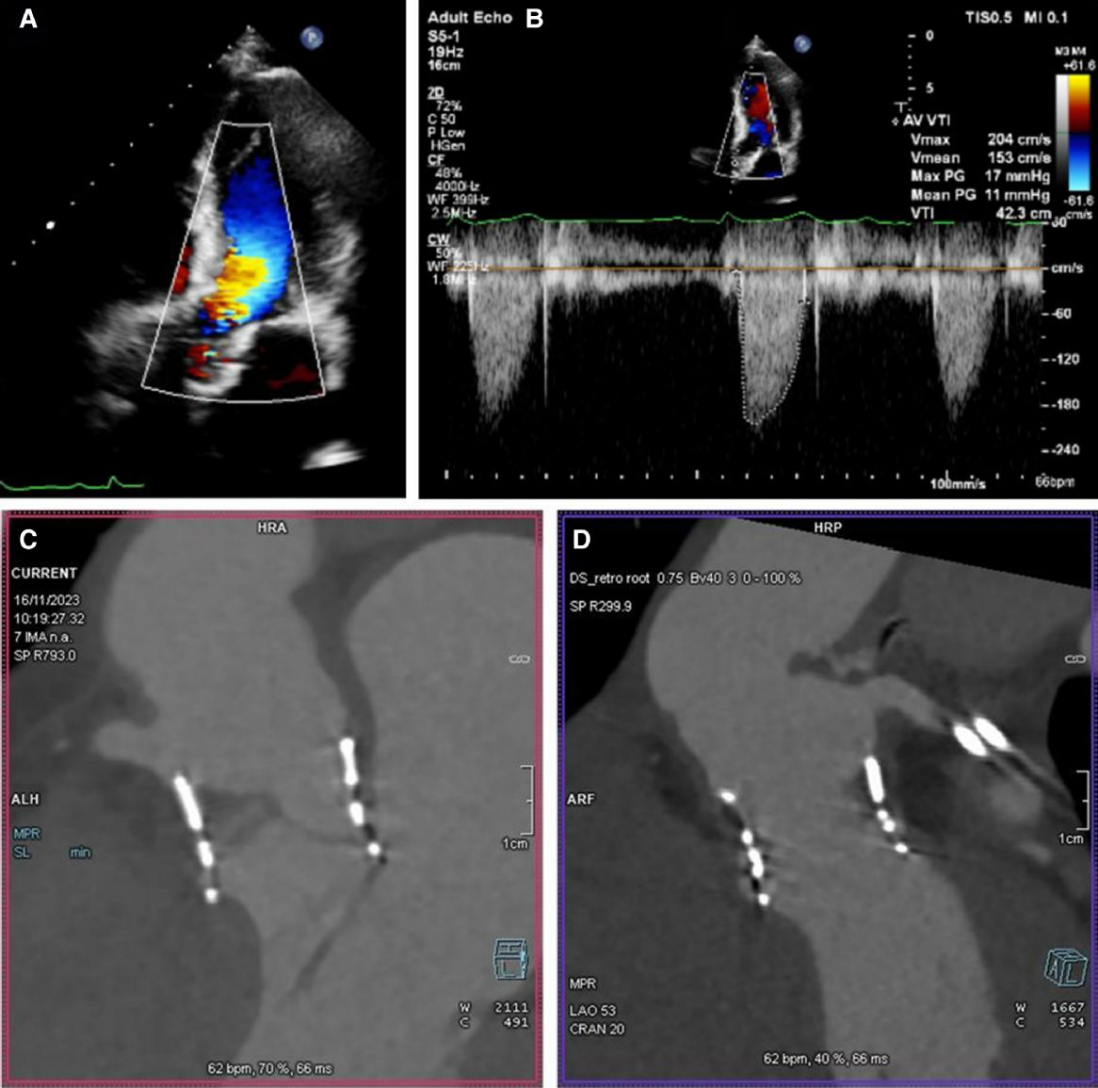
Transthoracic echocardiography 4 months post-transcatheter heart valve (THV) implantation: no residual aortic regurgitation by colour flow Doppler (*A*) and satisfactory transvalvular gradients (*B*); (*C* and *D*) CT scan demonstrating no migration of the THV and preservation of coronary access (left main coronary artery panel *C*, right coronary artery panel *D*).

In June 2023, via transfemoral access a 25 mm Edwards balloon was inflated within the aortic valve to guide THV sizing. The balloon anchored well with no waisting. In light of this, a 26 mm Sapien S3 Ultra with 4 cc overfill was chosen to achieve an area of ∼580 mm^2^ (estimated oversizing 10%). In view of the narrow coronary sinuses and STJ kink, the THV was positioned below the LMCA both to preserve immediate and future coronary access, should redo TAVR be required (*Figure 7*). There was no evidence of AR on aortography or echocardiography post-TAVR. ECG showed new left bundle branch block. TTE showed good valve parameters (Vmax 1.5 m/s; peak gradient 9 mmHg; mean gradient 4 mmHg and aortic valve area 2.2 cm^2^). Repeat TTE at 4 months found a stable, well-functioning THV with no paravalvular or transvalvular regurgitation. CT imaging at 8 months showed no migration of the THV and preserved coronary access (*Figure 8*).

## Discussion

VSARR is an effective treatment for aortic root dilatation and avoids the disadvantages of composite grafts with either mechanical or biological prostheses. However, VSARR failure with pure AR is an infrequent but recognized complication and poses significant challenges to treatment by TAVR due to frequent absence of leaflet calcification and significant risk of migration and embolization. There are little data to guide optimal sizing and choice of THV for this situation. In a previous case report of AR following VSARR, in the absence of calcification, treated with a BEV, oversizing of ∼30% was employed without aortic root injury or paravalvular regurgitation.^10^ However, the Dacron graft is relatively distensible and whilst a degree of oversizing is necessary for anchoring, such aggressive oversizing risks rupture at the suture line between the valsalva graft and LVOT. The David procedure (whereby the Dacron graft is sutured down to the level of the annulus) may provide more stability for THV anchoring than the Yacoub procedure.

This case series demonstrates the use of balloon-expandable THVs with modest oversizing only (10%–11%) to successfully manage pure AR occurring in VSARR failure in the absence of native leaflet calcification. Balloon-expanding valves may be preferable to self-expanding valves in this scenario due to the increased radial force for anchoring in the absence of calcification. Historically, studies have shown significantly reduced procedural success for off-label TAVR in AR without leaflet calcification with high rates of embolization/migration and residual paravalvular AR compared with TAVR in calcific aortic stenosis.^14^ In both cases presented here, positioning of the balloon-expandable THV was performed with a guide catheter in the left main stem (LMS), stabilized with a coronary dilatation balloon and without use of a pigtail catheter. Delineation of the LMS was felt to be better achieved with a catheter in the LMS than root aortography with a pigtail catheter. Both patients notably had a relatively narrow sinus of valsalva. As both patients were young, and potentially with life spans where redo TAVR was felt a possibility, we felt it important to ensure positioning of the balloon-expandable THV below the coronary ostia in order to allow unrestricted coronary access immediately and in the future should redo TAVR be required. This was felt particularly important in the first patient whose LMS height was very low at 7.1 mm.

Our series illustrates the importance of preprocedural planning and in particular careful analysis of the gated CT and use of an appropriately sized valvuloplasty balloon on which to help select appropriate THV sizing. We found stability of a mildly undersized valvuloplasty balloon reassuring and aimed for THV oversizing of 10%–11%. The mild degree of waisting seen on both THVs would further support this modest degree of oversizing to be adequate and neither patient suffered aortic root injury or residual AR with stability and absence of migration on interval CT at 3–8 months.

Although self-expanding supra-annular THVs have been successfully used in this situation,^13^ we prefer short-framed balloon-expandable valves in view of the better coronary access immediately^15^ and improved coronary access following redo TAVR.^16^

Notably, both patients were relatively young and who may require consideration of redo TAVR in the future and we found a guide catheter in the LMS at deployment very helpful in positioning the THV below the LMS.

Dedicated valves designed to treat AR may prove to be effective for this indication, such as the J valve (JC medical, California, USA) and the Trilogy valve, although these valves currently lack data for this use in this particular setting.

## Data Availability

All data are incorporated into the article.

## Lead author biography

Dr Sharma is an Interventional Cardiologist with an interest in structural heart intervention.

## Notes

**Consent:** The authors confirm that written consent for submission and publication of this case report including images and associated text has been obtained from the patient in line with COPE guidance.

**Funding:** No funding, grants, contracts or other form of financial support were relevant to this submission.

## References

[1] Sarsam MAI, Yacoub M. Remodeling of the aortic valve anulus. J Thorac Cardiovasc Surg 1993;105:435–438. 8445922

[2] David TE, Feindel CM. An aortic valve-sparing operation for patients with aortic incompetence and aneurysm of the ascending aorta. J Thorac Cardiovasc Surg 1992;103:617–622. 1532219

[3] Jahangiri M, Mani K, Acharya M, Bilkhu R, Quinton P, Schroeder F, et al Early and long-term outcomes of conventional and valve-sparing aortic root replacement. Heart 2022;108:1858–1863. 35580978 10.1136/heartjnl-2022-320870

[4] Kari FA, Doll KN, Hemmer W, Liebrich M, Sievers HH, Richardt D, et al Survival and freedom from aortic valve-related reoperation after valve-sparing aortic root replacement in 1015 patients. Interact Cardiovasc Thorac Surg 2016;22:431–438. 26718320 10.1093/icvts/ivv354

[5] Langer F, Aicher D, Kissinger A, Wendler O, Lausberg H, Fries R, et al Aortic valve repair using a differentiated surgical strategy. Circulation 2004;110:II67–II73. 15364841 10.1161/01.CIR.0000138383.01283.b8

[6] El Khoury G, Vanoverschelde JL, Glineur D, Poncelet A, Verhelst R, Astarci P, et al Repair of aortic valve prolapse: experience with 44 patients. Eur J Cardio-thoracic Surg 2004;26:628–633. 10.1016/j.ejcts.2004.05.027 15302061

[7] David TE, David CM, Ouzounian M, Feindel CM, Lafreniere-Roula M. A progress report on reimplantation of the aortic valve. J Thorac Cardiovasc Surg 2021;161:890–899.e1. 33008570 10.1016/j.jtcvs.2020.07.121

[8] Giebels C, Fister JC, Ehrlich T, Federspiel J, Schäfers HJ. Failures of valve-sparing aortic root replacement using the root remodeling technique. Ann Thorac Surg 2022;113:2000–2006. 34400134 10.1016/j.athoracsur.2021.07.034

[9] Patlolla SH, Saran N, Dearani JA, Stulak JM, Schaff HV, Greason KL, et al Outcomes and risk factors of late failure of valve-sparing aortic root replacement. J Thorac Cardiovasc Surg 2022;164:493–501.e1. 33077178 10.1016/j.jtcvs.2020.09.070

[10] Koren O, Patel V, Kaewkes D, Koseki K, Chakravarty T, Nakamura M, et al Transcatheter aortic valve replacement for bicuspid aortic insufficiency after valve-sparing aortic root replacement. JACC Case Rep 2021;3:1798–1802. 34917957 10.1016/j.jaccas.2021.07.018 PMC8642721

[11] Itaya N, ichiro SK, Takaseya T, Sasaki M, Yamaji K, Honda A, et al Transcatheter aortic valve implantation for aortic valve stenosis 17 years after aortic root remodeling via the Yacoub method. J Cardiol Cases 2023;27:287–289. 37283910 10.1016/j.jccase.2023.02.018 PMC10240408

[12] Favero L, De Leo A, Daniotti A, Calzolari D, Gasparetto N, Minniti G, et al Transfemoral transcatheter aortic valve implantation for treatment of severe aortic regurgitation in a patient with previous aortic valve-sparing operation according to David. Cardiovasc Revascularization Med 2017;18:611–615. 10.1016/j.carrev.2017.06.010 28779858

[13] Tay ELW, Kong WKF, Yip JWL, Low TT, Hon JKF. Successful transcatheter aortic valve replacement for severe aortic valve regurgitation following a David I valve-sparing procedure. JACC Cardiovasc Interv 2017;10:e101–e103. 28527766 10.1016/j.jcin.2017.03.035

[14] Poletti E, De Backer O, Scotti A, Costa G, Bruno F, Fiorina C, et al Transcatheter aortic valve replacement for pure native aortic valve regurgitation: the PANTHEON international project. JACC Cardiovasc Interv 2023;16:1974–1985. 37648345 10.1016/j.jcin.2023.07.026

[15] Tarantini G, Nai Fovino L, Scotti A, Massussi M, Cardaioli F, Rodinò G, et al Coronary access after transcatheter aortic valve replacement with commissural alignment: the ALIGN-ACCESS study. Circ Cardiovasc Interv 2022;15:E011045. 35167332 10.1161/CIRCINTERVENTIONS.121.011045

[16] Buzzatti N, Montorfano M, Romano V, De Backer O, Soendergaard L, Rosseel L, et al A computed tomography study of coronary access and coronary obstruction after redo transcatheter aortic valve implantation. EuroIntervention 2020;16:E1005–E1013. 32928715 10.4244/EIJ-D-20-00475

